# Poor agreement between interferon-gamma release assays and the tuberculin skin test among HIV-infected individuals in the country of Georgia

**DOI:** 10.1186/1471-2334-13-513

**Published:** 2013-11-01

**Authors:** Nikoloz Chkhartishvili, Russell R Kempker, Natia Dvali, Lela Abashidze, Lali Sharavdze, Pati Gabunia, Henry M Blumberg, Carlos del Rio, Tengiz Tsertsvadze

**Affiliations:** 1 Infectious Diseases, AIDS and Clinical Immunology Research Center, 16 Al. Kazbegi Avenue, Tbilisi, 0160, Georgia; 2 Division of Infectious Diseases, Emory University School of Medicine, 1364 Clifton Road, Atlanta, Georgia, 30322, , USA; 3 Tbilisi State University Faculty of Medicine, 16 Al. Kazbegi Avenue, Tbilisi, 0160, Georgia; 4 Hubert Department of Global Health, Emory University Rollins School of Public Health, 1518 Clifton Road, Atlanta, Georgia, 30322, , USA; 5 Center for AIDS Research, Emory University, 1518 Clifton Road, Atlanta, Georgia, 30322, USA

**Keywords:** Latent tuberculosis infection, Screening, TST, Interferon-gamma, Eastern Europe

## Abstract

**Background:**

Improved tests to diagnose latent TB infection (LTBI) are needed. We sought to evaluate the performance of two commercially available interferon-gamma release assays (IGRAs) compared to the tuberculin skin test (TST) for the diagnosis of LTBI and to identify risk factors for LTBI among HIV-infected individuals in Georgia, a country with high rates of TB.

**Methods:**

HIV-patients were enrolled from the National AIDS Center in Tbilisi, Georgia. After providing informed consent, each participant completed a questionnaire, had blood drawn for QuantiFERON-TB Gold in-Tube (QFT-GIT) and T-SPOT.TB testing and had a TST placed. The TST was read at 48–72 hrs with ≥ 5 mm induration considered positive.

**Results:**

Between 2009–2011, 240 HIV-infected persons (66% male) with a median age of 38 years and a median CD4 count of 255 cells/μl (IQR: 124–412) had diagnostic testing for LTBI performed. 94% had visible evidence of a BCG scar. The TST was positive in 41 (17%) patients; QFT-GIT in 70 (29%); and T-SPOT.TB in 56 (24%). At least one diagnostic test was positive in 109 (45%) patients and only among 13 (5%) patients were all three tests positive. Three (1%) QFT-GIT and 19 (8%) T-SPOT.TB test results were indeterminate. The agreement among all pairs of tests was poor: QFT-GIT vs. T-SPOT.TB (κ = 0.18, 95% CI .07-.30), QFT-GIT vs. TST (κ = 0.29, 95% CI .16-.42), and TST vs. T-SPOT.TB (κ = 0.22, 95% CI .07-.29). Risk factors for LTBI varied by diagnostic test and none showed associations between positive test results and well-known risk factors for TB, such as imprisonment, drug abuse and immunological status.

**Conclusions:**

A high proportion of HIV patients had at least one positive diagnostic test for LTBI; however, there was very poor agreement among all tests. This lack of agreement makes it difficult to know which test is superior and most appropriate for LTBI testing among HIV-infected patients. While further follow-up studies will help determine the predictive ability of different LTBI tests, improved modalities are needed for accurate detection of LTBI and assessment of risk of developing active TB among HIV-infected patients.

## Background

HIV is the greatest risk factor for progression of recent or latent tuberculosis infection (LTBI) to active tuberculosis (TB) disease. The risk of developing active TB is more than 20 times greater in HIV patients as compared to immunocompetent persons [1,2]. Given this extremely high risk, the accurate diagnosis and subsequent treatment of LTBI among persons living with HIV (PLHIV) is regarded as an essential component of TB control strategy [3,4]. Yet, the best available diagnostic tools for LTBI are not fully defined.

T-cell based interferon-gamma release assays (IGRA) offer several advantages over the tuberculin skin test (TST), including better specificity (especially among those with Bacille Calmette-Guérin [BCG] vaccination), elimination of the subjectivity of TST reading, and logistic convenience [5,6]. However, the data comparing IGRAs and the TST in immunocompromised persons is limited and shows no clear superiority of one test over another [7].

Following the collapse of Soviet Union, the country of Georgia experienced significant socio-economic upheavals resulting in a deterioration of public health infrastructure and resurgence of TB in the 1990s. TB incidence rates increased from 28/100,000 to 186/100,000 between 1990 and 1997 and continue to remain high at 125 TB cases per 100,000 population in 2011 [8]. While Georgia has been able to avoid a large-scale HIV epidemic, 3,642 HIV cases have been reported since 1989. The estimated adult HIV prevalence in Georgia is 0.2%, [9] but the number of reported HIV cases has been steadily increasing. Similar to other Eastern European countries the HIV epidemic in Georgia has been driven by injection drug use (IDU) accounting for 54% of total reported cases. HIV, substance abuse, incarceration and low socioeconomic status are well-known risk factors for TB, [10-12] which may contribute to the significant impact of TB among PLHIV in Georgia. Data from the national HIV/AIDS clinical program found that 20% of registered HIV patients had received a diagnosis of TB, and that TB was responsible for 25% of all deaths among PLHIV in the country [13].

Addressing the TB/HIV co infection has become a country health priority and a national TB/HIV strategic plan was developed in 2007. While there is well established collaborative network ensuring free access to both TB and HIV medical care in Georgia the diagnosis and treatment of LTBI among PLHIV needs to be scaled-up. The objectives of the present study were to assess the performance of two commercially available IGRAs (QuantiFERON-TB Gold in Tube [QFT-GIT] and T-SPOT.TB [TSPOT]) compared to the TST for the diagnosis of LTBI in HIV-infected patients, and to identify risk factors for LTBI in effort to improve the TB prevention and care among PLHIV in Georgia.

## Methods

### Study setting and population

A cross-sectional study was conducted at the Infectious Diseases, AIDS and Clinical Immunology Research Center (IDACIRC) in Tbilisi, Georgia between November 2009 and June 2011. The IDACIRC is the national referral institution for HIV diagnosis, treatment and care. Inclusion criteria for study enrollment included age ≥18 years old, confirmed HIV infection, and ability to provide written informed consent. Patients with a history of active TB disease were excluded. After informed consent, all participants completed a study questionnaire, and were tested for LTBI using the IGRAs and TST. Blood was drawn for the IGRAs prior to the placement of the TST.

All patients were interviewed to collect information regarding socio-demographic characteristics, history of BCG vaccination, imprisonment, tobacco use and substance abuse. Patients were screened for illicit drug use and alcohol abuse using the Drug Abuse Screening Test (DAST-10) [14] and the Alcohol Use Disorders Identification Test (AUDIT) [15] respectively. Additionally, medical chart abstraction was performed to collect the following information: most recent CD4+ T-cell count, HIV-1 viral load, hepatitis B virus (HBV) and hepatitis C virus (HCV) status, and antiretroviral therapy (ART) use.

The study was approved by the IDACIRC and Emory University institutional review boards (IRBs).

### TST and IGRA assays

The TST was performed using the Mantoux method. An intradermal injection of 0.1 ml purified protein derivative was administered into the volar surface of the forearm. The transverse diameter of induration was recorded in millimeters 48–72 hours after administration. An induration of ≥ 5 mm of induration was considered positive among the HIV-infected persons included in this study [16]. Each participant had approximately 12 ml of blood drawn for the QFT-GIT and TSPOT, which were performed according to the manufacturer’s instructions. Both the QFT-GIT and TSPOT were performed at the IDACIRC laboratory.

As recommended by the manufacturer and the U.S. Centers for Disease Control and Prevention (CDC), [17] the QFT-GIT result was considered positive if the interferon-gamma response to TB antigens minus the negative control was ≥ 0.35 IU/ml and also > 25% of the negative control; negative if these criteria were not met; and indeterminate if either the negative control had a result of > 8 IU/ml or the positive control had a result of < 0.5 IU/ml. For TSPOT 250,000 peripheral blood mononuclear cells (PBMCs) were isolated and plated per well: a nil control, a positive control containing phytohemagglutinin and TB specific antigens (CFP-10 and ESAT-6). Spot forming units were counted using AID EliSpot Reader System (Autoimmun Diagnostika, Germany). The test result was considered reactive if the response to either CFP-10 or ESAT-6 minus the nil control was ≥ 6 spot forming cells, or twice the nil control. The result was considered indeterminate if nil control spot count was > 10 spot forming cells or if the reading in the positive control was < 20 spot forming cells.

### Statistical analysis

Study data were collected and managed using REDCap electronic data capture tools hosted at Emory University [18]. All statistical analyses were performed using SAS 9.2 (SAS Institute, Cary, NC USA). Distributions of outcome variables and covariates were evaluated in descriptive statistics. The impact of immunosuppression as measured by the CD4+ T-cell count (<100, 100–200, >200 cells/uL) on LTBI test results was studied using a stratified analysis. Agreement between the three LTBI tests was evaluated using kappa (k) statistic, where κ > 0.75 represents excellent agreement, κ = 0.4-0.75 represents fair to good agreement, and κ <0.4 represents poor agreement [19]. Univariate logistic regression analysis was performed to identify risk factors associated with a positive LTBI test result. A purposeful variable selection strategy was used to build the final multivariate logistic regression models [20]. Criterion for retaining variables in the model was set at *p* value of 0.10. Confounding was assessed as 20% change in parameter estimate. A p value <0.05 was considered statistically significant.

## Results

### Study population

A total of 240 HIV-infected patients were enrolled in the study (Table 1). The median age was 38 years (range 33 – 44) and 66% were male. Nearly one in five (19%) patients had a history of imprisonment and 46% were co-infected with hepatitis C virus (HCV). The median CD4+ T-cell count of study participants was 255 cells/μl and 62 (26%) were receiving antiretroviral therapy (ART) for a median duration of 3 months. Visible evidence of BCG scar was present in 94% of patients. With regard to substance use, 63% of patients were current tobacco users, 13% had medium to high level of alcohol consumption as measured by the AUDIT screen, and 33% reported a medium to severe level of drug abuse by DAST screen.

**Table 1:** Patient characteristics of HIV-infected subjects undergoing latent tuberculosis testing (n = 240)

**Characteristic**	**All subjects n (%)**
Male	159 (66%)
Age, median (IQR)	38.0 (32.8-43.8)
High school education or less	124 (52%)
Unemployed	172 (72%)
Married	116 (48%)
History of imprisonment	46 (19%)
Household members, median (IQR)	3.0 (3.0-4.0)
**HIV related factors**	
On ART, at enrollment median time on ART (months)	62 (26%) 3
CD4 Count, median (IQR)	255 (124–412)
HIV RNA < 75 copies/ml	28 (12%)
Hepatitis C antibody positive	111 (46%)
Hepatitis B surface antigen positive	9 (4%)
**TB related factors**	
Household member with prior TB treatment	13 (5%)
Self reported BCG vaccination	173 (72%)
BCG scar	219 (94%)
**Drug use**	
Current tobacco smokers	152 (63%)
Alcohol abuse measure (per AUDIT score)	
< 8	208 (87%)
8-15	25 (10%)
16-19	2 (1%)
>20	5 (2%)
Drug abuse measure (per DAST score)	
0	147 (61%)
1-2	13 (5%)
3-5	27 (11%)
6-8	39 (16%)
9-10	14 (6%)

### LTBI test results

Among the 240 study participants, 109 (45%) had at least one positive test result. The prevalence of a positive TST was 17%, QFT-GIT 29%, and TSPOT 24% (Table 2). There were significantly more indeterminate TSPOT test results as compared to the QFT-GIT and TST (8% vs. 1% vs. 1%, P < 0.05). There were more positive test results using the IGRAs compared to the TST among patients with CD4 count <200 cells/μl, with difference between TSPOT and TST reaching statistical significance (TSPOT 29% vs. TST 16%, p = 0.01 and QFT-GIT 25% vs. TST 16%, p = 0.10). Overall,There were also more positive QFT-GIT and TSPOT test results as compared to the TST among patients with CD4 counts < 100 cells/μl, but the differences did not reach statistical significance (26% vs. 26% vs. 13%, respectively, p = 0.12).

**Table 2:** Overall TST, QFT-GIT, and T.SPOT results and per CD4 category

**Test results**	**CD4 count categories**			**Overall n = 240 (%)**
	**<100 n = 54 (%)**	**100-200 n = 37 (%)**	**>200 n = 149 (%)**	
**TST results**				
TST +	7 (13)	8 (22)	26 (18)	41 (17)
TST -	47 (87)	29 (78)	121 (82)	197 (83)
Indeterminate*	0	0	2 (1)	2 (1)
**QFT-GIT results**				
QFT +	14 (26)	9 (24)	47 (31)	70 (29)
QFT -	38 (70)	28 (76)	101 (68)	167 (70)
Indeterminate	2 (4)	-	1 (1)	3 (1)
**T.SPOT results**				
T.SPOT +	14 (26)	12 (32)	30 (20)	56 (24)
T.SPOT -	34 (64)	20 (54)	108 (73)	162 (68)
Indeterminate	5 (10)	5 (14)	10 (7)	20 (8)

* Either patient did not come to have read or returned > 72 hours after TST placement.

**Figure 1:**
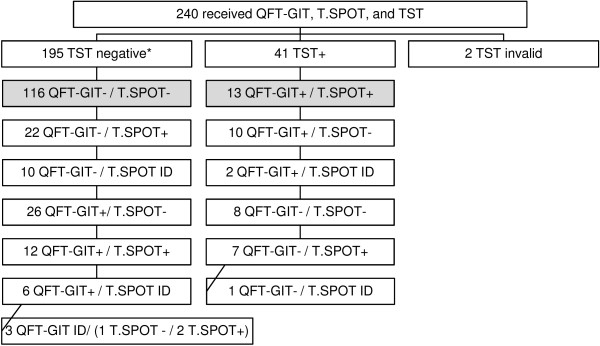
**Flow diagram for HIV-infected persons who underwent QFT-GIT, T.SPOT and TST.** *2 QFT-GIT and T.SPOT results missing. Shaded cells indicate agreement between all three tests. ID = indeterminate.

The overall concordance among the tests was poor; all three test results were in agreement only 54% of the time (129/240) (Figure 1). Only 13 (5%) patients had a positive result for all three LTBI diagnostic tests (TST, QFT-GIT, and TSPOT) and 116 (48%) patients had a negative test result for all three diagnostic tests. Two TST results were invalid and two IGRA results were missing. As measured by the Kappa statistic and shown in Table 3 the agreement between any two LTBI tests was poor: QFT-GIT vs. TSPOT k = 0.18 (95% CI: 0.07-0.30), QFT-GIT vs. TST k = 0.29 (95% CI: 0.16-0.42), TST vs. TSPOT k = 0.22 (95% CI: 0.07-0.29).

**Table 3:** Agreement between TST, QFT-GIT and T-SPOT.TB

	**Kappa (95% CI)**
QFT-GIT vs. TSPOT	0.18 (95% CI: 0.07-0.30)
QFT-GIT vs. TST	0.29 (95% CI: 0.16-0.42)
TST vs. TSPOT	0.22 (95% CI: 0.07-0.29)

In comparing quantitative QFT-GIT results stratified by TST and TSPOT test results, we found higher median and mean QFT-GIT results in patients with a positive TST as compared to a negative TST (Table 4). There was no significant difference of mean and median QFT-GIT results between TSPOT positive and negative patients. Regardless of the TST result, the mean QFT-GIT response was lower among patients with indeterminate TSPOT results compared to either positive or negative TSPOT results.

**Table 4:** Median and average QFT-GIT test result values for different combination of TST and T-SPOT.TB test results

**TST -**	**QFT-GIT median**	**IQR**	**TST +**	**QFT-GIT median**	**IQR**
26 QFT-GIT +/T-SPOT -	1.56	0.57-3.0	10 QFT-GIT +/T.SPOT -	2.18	1.01-9.25
12 QFT-GIT +/T-SPOT +	0.74	0.56-1.97	13 QFT-GIT +/T.SPOT +	3.46	1.17-4.41
6 QFT-GIT +/T-SPOT ID	1.33	0.64-1.87	2 QFT-GIT +/ T.SPOT ID	1.55	0.56-2.54

### Risk factors for positive LTBI test

The results of univariate and multivariate logistic regression analyses evaluating risk factors for a positive LTBI test result are shown in Table 5. Risk factors were assessed separately for each diagnostic test. None of the well-known risk factors for TB, such as imprisonment, drug abuse and immunological status were associated with positive test results. In multivariate analysis HCV co-infection (aOR 2.18, 95% CI 1.01-4.71) and receiving ART (aOR 0.15, 95% CI 0.04-0.52) were significantly associated with a positive TST result. Male gender was the only risk factor significantly associated with a positive QFT test (aOR 2.92. 95% CI 1.49-5.74). Increasing age per year (aOR 1.04, 95% CI 1.002-1.08) and chronic hepatitis B infection (aOR 5.13, 95% CI 1.24-21.17) were the only factors significantly associated with a positive T-SPOT.TB test in multivariate analysis.

**Table 5:** Logistic regression analysis of association of risk factors for LTBI with a positive TST, QFT-GIT, and T-SPOT.TB result

**Risk factors**	**Positive TST result**				**Positive QFT-GIT**				**Positive T-SPOT.TB**			
	**n = 41**				**n = 70**				**n = 56**			
	**Univariate**		**Multivariate**		**Univariate**		**Multivariate**		**Univariate**		**Multivariate**	
	**OR**	**95% CI**	**OR**	**95% CI**	**OR**	**95% CI**	**OR**	**95% CI**	**OR**	**p**	**OR**	**95% CI**
Male	1.75	0.81-3.77			2.92	1.49-5.75	2.92	1.49-5.75	1.93	0.97-3.84	1.82	0.90-3.67
Age (per year)	1.01	0.97-1.05			1.01	0.97-1.04			1.04	1.004-1.08	1.04	1.003-1.08
Imprisonment	2.31	1.08-4.92	2.09	0.88-4.97	2.22	1.14-4.31			1.78	0.88-3.61		
Unemployment	0.80	0.38-1.69			1.22	0.63-2.34			1.15	0.57-2.33		
On ART	0.19	0.06-0.62	0.15	0.04-0.52	0.80	0.42-1.53			0.66	0.32-1.39		
VL <75	0.34	0.08-1.48			0.63	0.24-1.63			1.10	0.44-2.73		
CD4 <100	-	-			-	-			-	-		
100 < CD4 <200	1.85	0.61-5.65			0.92	0.35-2.42			1.34	0.53-3.36		
CD4 >200	1.44	0.59-3.55			1.32	0.66-2.65			0.71	0.34-1.47		
Hepatitis C Ab +	2.38	1.19-4.77	2.18	1.01-4.71	1.72	0.98-3.00			1.78	0.97-3.26		
Hepatitis B sAG +	1.39	0.28-6.96			2.00	0.52-7.68			4.36	1.13-16.85	5.13	1.24-21.17
Household Member treated for TB	1.48	0.39-5.62			0.43	0.09-1.97			1.48	0.44-5.00		
BCG	2.55	0.32-20.18			1.41	0.38-5.29			1.78	0.38-8.28		
Tobacco	1.32	0.65-2.71			2.02	1.09-3.75			1.16	0.62-2.18		
ETOH (AUDIT > = 8)	1.42	0.57-3.54			0.94	0.41-2.16			1.87	0.84-4.17		
Drug Use (DART > =3)	2.02	1.02-4.01			2.33	1.31-4.16			1.42	0.76-2.64		

## Discussion

We found that a high proportion of HIV-infected patients in the country of Georgia had at least one positive LTBI test result (45%) with either the TST, QFT-GIT, or TSPOT assay. The higher proportion of positive IGRA test results as compared to the TST was most pronounced among patients with CD4 counts ≤ 100 μl, suggesting the IGRAs may perform better in highly immunocompromised patients. However, the lack of a gold standard for the diagnosis of LTBI, scarcity of data regarding the long term predictive value of IGRAs, and the very poor agreement among the three tests makes it unclear which test is optimal. While it is unclear which LTBI test performed best, our study does demonstrate that LTBI is common among HIV infected patients in Georgia and is an urgent problem that needs addressing.

The diagnosis and treatment of LTBI is a key component of the WHO three I’s program for decreasing the impact of TB among HIV-infected persons [3,4]. Accurate identification of patients with LTBI remains challenging. In the absence of gold standard, agreement between tests serves as surrogate marker for performance. Our study showed poor agreement both between IGRAs and TST, and between the two IGRAs. Agreement was especially low between QFT-GIT and TSPOT (k = 0.18), which is similar to other reports [21-23]. Some studies have reported better agreement, but never surpassing moderate levels [24,25]. The reason for discordance between the two IGRAs in our patient population is unclear. Additionally, we found no difference in quantitative QFT-GIT values based on TSPOT results further confirming the discordance between the two tests. Indeterminate results were more common with T.SPOT (8%) compared to QFT (1%). In our study, indeterminate results did not seem to be associated with degree of immunodeficiency as seen elsewhere [22,26,27]. Given the poor concordance between diagnostic tests, our study suggests the urgent need for new and better diagnostic tests for LTBI, especially among HIV-infected persons who are at greatest risk for progression to active TB disease following infection.

The proportion of patients with positive test results among those with severe immunodeficiency (CD4 count <100 cells/μl) was higher with IGRAs as compared to the TST (26% vs. 13%, p = 0.12) but the differences were not statistically significant. The TSPOT and QFT-GIT also yielded higher proportions of positive test results that the TST among patients with CD4 count <200 cells/μl (TSPOT 29% vs. TST 16%, p = 0.01 and QFT-GIT 25% vs. TST 16%, p = 0.10). Additionally, in contrast to prior studies, reporting significantly lower proportion of positive IGRA test results in patients with CD4 count <200cell/μl, in our study both the QFT-GIT and TSPOT had similar proportions of positive test results for patients above and below a CD4 count of 200 cells/μl [7]. Some authors have suggested, [25,28] that the IGRAs are more sensitive for detection infection with *M. tuberculosis* in immunosuppressed patients than TST. However, absence of gold standard makes it difficult to conclude whether IGRAs outperformed TST, or if there is a higher rate of false positive results. One recent study found a high rate of positive QFT-GIT tests that reverted to negative upon repeat testing in low risk HIV-infected patients [29]. The reversion rate was much higher in American born HIV-infected patients (80%) as compared to patients originally from high incidence TB countries (25%), such as Georgia.

Multivariate analysis of risk factors for LTBI showed heterogeneity across diagnostic tests. Positive TST was associated with co-infection with hepatitis C and being on ART (protective effect), male gender was associated with positive QFT-GIT test, and increasing age together with chronic hepatitis B infection were significantly associated with positive TSPOT result. Well known risk factors for tuberculosis, such as imprisonment and drug abuse, [30] were associated with the outcome only in univariate analysis, but not in multivariate. Association of viral hepatitis co-infection with TST and TSPOT positivity merits further exploration.

This study has several limitations. Although our study sample size is comparable to previous reports, we had a relatively small number of HIV-infected patients with low CD4 counts. Our study was cross sectional so there was no patient follow up for the development of active tuberculosis. This prohibited us from evaluating the predictive value of IGRAs for the development of active tuberculosis among HIV-infected patients. Further studies are needed to assess the predictive value of IGRAs for active TB, especially among immunocompromised patients such as those with HIV infection [31-33].

There remains uncertainty about which is the best diagnostic test for LTBI among HIV-infected persons. Despite the uncertainty, a growing number of guidelines support the use of IGRA for the diagnosis of LTBI (either in combination with TST or alone) [34]. In addition, recent ART guidelines from the WHO Regional Office for Europe identifies IGRAs as preferred diagnostic method for LTBI screening in HIV patients WHO does not support the use of IGRAs in low and middle income countries, [35] Given the poor concordance among the three diagnostic tests (and between the two commercially available IGRAs), our data supports the WHO recommendations regarding the use of these diagnostic tests in low and middle income countries. Recent U.S. CDC guidelines recommend use IGRAs in persons with BCG vaccination and those with low rates of returning to have TST read [17]. The CDC and Canadian Tuberculosis Committee (CTC) guidelines also discuss the possible utility of dual testing with IGRAs and TST for LTBI among high-risk individuals [17,36]. The CTC specifically recommends performing an IGRA in immunocompromised individuals with a strong suspicion for LTBI if the initial TST is negative. If this strategy was used for our patient cohort an additional 44 patients and 36 patients would have been diagnosed with LTBI by the QFT-GIT and TSPOT tests respectively. Given the varying performance and agreements of LTBI tests across different settings it is likely that different strategies will be needed depending on the population. Additional factors that need to be taken into consideration included patients preferences, logistics, and test cost. The cost of a single IGRA may be up to three times as a high as the cost of a TST [37,38].

## Conclusion

In summary, we report the first study to evaluate performance of three diagnostic tests for LTBI in HIV patients in the Eastern European region. While our study showed a high prevalence of LTBI we also found a poor concordance between all LTBI diagnostic tests (QFT-GIT, TSPOT, and TST) including between the two different commercially available IGRAs. Multivariate analysis did not identify one specific population sub-group at higher risk of LTBI. Variation in risk factors for LTBI across the tests reflects poor agreement between available diagnostic modalities. This lack of agreement makes it difficult to identify most appropriate test for LTBI diagnosis among HIV-infected patients. Without clear evidence of superiority of IGRAs, choosing test for LTBI, particularly in resource-limited settings, should account for costs and logistics. While long-term follow-up studies will help to better understand the role of IGRAs among HIV infected patients, improved modalities are needed to accurately identify HIV-infected patient at highest risk of developing active TB, who will benefit the most from LTBI treatment.

## Abbreviations

HIV: Human immunodeficiency virus; AIDS: Acquired immunodeficiency syndrome; TB: Tuberculosis; LTBI: Latent tuberculosis infection; BCG: Bacillus calmette–guérin; TST: Tuberculin skin test; IGRA: Interferon-gamma release assay; QFT-GIT: QuantiFERON-TB gold in-tube assay.

## Competing interests

The authors declare that they have no competing interests.

## Author’s contributions

NC contributed to study design, data management, also drafted and revised the manuscript. RRK was responsible for data management and analysis, and revised the manuscript. ND and LA performed all interferon-gamma release assays and contributed to the methods section of the manuscript. LS and PG were responsible for enrollment, data collection and oversaw performance of TST. HMB contributed to concept and study design, also critically reviewed and edited the manuscript. CDR and TT contributed to the concept and study design, provided oversight of the study and edited the manuscript. All authors read and approved the final manuscript.

## Pre-publication history

The pre-publication history for this paper can be accessed here:

http://www.biomedcentral.com/1471-2334/13/513/prepub

## References

[B1] Selwyn P Hartel D Lewis V Schoenbaum E Vermund S Klein R Walker A Friedland G A prospective study of the risk of tuberculosis among intravenous drug users with human immunodeficiency virus infection N Engl J Med 1989 320 545 550 10.1056/NEJM198903023200901 2915665

[B2] Allen S Batungwanayo J Kerlikowske K Lifson A Wolf W Granich R Taelman H Van de Perre P Serufilira A Bogaerts J Two-year incidence of tuberculosis in cohorts of HIV-infected and uninfected urban Rwandan women Am Rev Respir Dis 1992 146 1439 1444 10.1164/ajrccm/146.6.1439 1456559

[B3] Taylor Z Nolan CM Blumberg HM Controlling tuberculosis in the United States: recommendations from the American thoracic society, CDC, and the infectious diseases society of America MMWR Recomm Rep 2005 54 1 81 16267499

[B4] World Health Organization Guidelines for Intensified Tuberculosis Case-Finding and Isoniazid Preventive Therapy for People Living with HIV in Resource-Constrained Settings 2011 Geneva: World Health Organization

[B5] Whalen CC Diagnosis of latent tuberculosis infection: measure for measure JAMA 2005 293 2785 2787 10.1001/jama.293.22.2785 15941809

[B6] Pai M Zwerling A Menzies D Systematic review: T-cell-based assays for the diagnosis of latent tuberculosis infection: an update Ann Intern Med 2008 149 177 184 10.7326/0003-4819-149-3-200808050-00241 18593687 PMC2951987

[B7] Cattamanchi A Smith R Steingart KR Metcalfe JZ Date A Coleman C Marston BJ Huang L Hopewell PC Pai M Interferon-gamma release assays for the diagnosis of latent tuberculosis infection in HIV-infected individuals: a systematic review and meta-analysis J Acquir Immune Defic Syndr 2011 56 230 238 10.1097/QAI.0b013e31820b07ab 21239993 PMC3383328

[B8] WHO Regional Office for Europe European Health for All Database (HFA-DB): Tuberculosis Incidence 2012 Copenhagen: WHO Regional Office for Europe http://data.euro.who.int/hfadb/

[B9] UNAIDS Global Report: UNAIDS Report on the Global AIDS Epidemic 2010 2010 Geneva: UNAIDS

[B10] Garfein RS Lozada R Liu L Laniado-Laborin R Rodwell TC Deiss R Alvelais J Catanzaro A Chiles PG Strathdee SA High prevalence of latent tuberculosis infection among injection drug users in Tijuana, Mexico Int J Tuberc Lung Dis 2009 13 626 632 19383197 PMC2744313

[B11] Martin V de Garcia Olalla P Orcau A Cayla JA Factors associated with tuberculosis as an AIDS-defining disease in an immigration setting J Epidemiol 2011 21 108 113 10.2188/jea.JE20100072 21325728 PMC3899502

[B12] Menzies D Baussano I Williams BG Nunn P Beggiato M Fedeli U Scano F Tuberculosis incidence in prisons: a systematic review PLoS Med 2010 7 e1000381 10.1371/journal.pmed.1000381 21203587 PMC3006353

[B13] Tsertsvadze T Chkhartishvili N Gamkrelidze A Rukhadze N Bolokadze N Gabunia P Sharvadze L Analysis of Causes of Death among HIV-Infected Persons in Georgia, 1989–2009 2010 Vienna: XVIII International AIDS Conference Abstract no. TUPE0225

[B14] Skinner HA The drug abuse screening test Addict Behav 1982 7 363 371 10.1016/0306-4603(82)90005-3 7183189

[B15] Babor TF Higgins-Biddle JC Saunders JB Monteiro MG AUDIT: The Alcohol Use Disorders Identification Test: Guidelines for Use in Primary Health Care 2001 2 Geneva: World Health Organization

[B16] Centers for Disease Control and Prevention Targeted tuberculin testing and treatment of latent tuberculosis infection: American thoracic society MMWR Recomm Rep 2000 49 1 51 10881762

[B17] Mazurek GH Jereb J Vernon A LoBue P Goldberg S Castro K Updated guidelines for using interferon gamma release assays to detect mycobacterium tuberculosis infection - United States, 2010 MMWR Recomm Rep 2010 59 1 25 20577159

[B18] Harris PA Taylor R Thielke R Payne J Gonzalez N Conde JG Research electronic data capture (REDCap)–a metadata-driven methodology and workflow process for providing translational research informatics support J Biomed Inform 2009 42 377 381 10.1016/j.jbi.2008.08.010 18929686 PMC2700030

[B19] Kraemer HC Measurement of reliability for categorical data in medical research Stat Methods Med Res 1992 1 183 199 10.1177/096228029200100204 1341657

[B20] Bursac Z Gauss CH Williams DK Hosmer D Purposeful selection of variables in logistic regression Source Code Biol Med 2008 3 17 10.1186/1751-0473-3-17 19087314 PMC2633005

[B21] Leidl L Mayanja-Kizza H Sotgiu G Baseke J Ernst M Hirsch C Goletti D Toossi Z Lange C Relationship of immunodiagnostic assays for tuberculosis and numbers of circulating CD4+ T-cells in HIV infection Eur Respir J 2009 35 619 626 19608590 10.1183/09031936.00045509

[B22] Talati NJ Seybold U Humphrey B Aina A Tapia J Weinfurter P Albalak R Blumberg HM Poor concordance between interferon-gamma release assays and tuberculin skin tests in diagnosis of latent tuberculosis infection among HIV-infected individuals BMC Infect Dis 2009 9 15 10.1186/1471-2334-9-15 19208218 PMC2649136

[B23] Stephan C Wolf T Goetsch U Bellinger O Nisius G Oremek G Rakus Z Gottschalk R Stark S Brodt H-R Staszewski S Comparing QuantiFERON-tuberculosis gold, T-SPOT tuberculosis and tuberculin skin test in HIV-infected individuals from a low prevalence tuberculosis country AIDS 2008 22 2471 2479 10.1097/QAD.0b013e3283188415 19005270

[B24] Mandalakas AM Hesseling AC Chegou NN Kirchner HL Zhu X Marais BJ Black GF Beyers N Walzl G High level of discordant IGRA results in HIV-infected adults and children Int J Tuberc Lung Dis 2008 12 417 423 18371268

[B25] Talati NJ Gonzalez-Diaz E Mutemba C Wendt J Kilembe W Mwananyanda L Chomba E Allen S Del Rio C Blumberg HM Diagnosis of latent tuberculosis infection among HIV discordant partners using interferon gamma release assays BMC Infect Dis 2011 11 264 10.1186/1471-2334-11-264 21962029 PMC3198954

[B26] Oni T Gideon HP Bangani N Tsekela R Seldon R Wood K Wilkinson KA Goliath RT Ottenhoff TH Wilkinson RJ Risk factors associated with indeterminate interferon gamma responses in the assessment of latent tuberculosis infection in a high incidence environment Clin Vaccine Immunol 2012 19 1243 1247 10.1128/CVI.00166-12 22718129 PMC3416070

[B27] Weinfurter P Blumberg HM Goldbaum G Royce R Pang J Tapia J Bethel J Mazurek GH Toney S Albalak R Predictors of discordant tuberculin skin test and QuantiFERON®-TB Gold In-Tube results in various high-risk groups Int J Tuberc Lung Dis 2011 15 1056 1061 10.5588/ijtld.10.0650 21740668

[B28] Balcells ME Pérez CM Chanqueo L Lasso M Villanueva M Espinoza M Villarroel L García P A comparative study of two different methods for the detection of latent tuberculosis in HIV-positive individuals in Chile Int J Infect Dis 2008 12 645 652 10.1016/j.ijid.2008.03.005 18534887

[B29] Gray J Reves R Johnson S Belknap R Identification of false-positive QuantiFERON-TB Gold In-Tube assays by repeat testing in HIV-infected patients at low risk for tuberculosis Clin Infect Dis 2012 54 e20 23 10.1093/cid/cir792 22057704

[B30] Getahun H Gunneberg C Sculier D Verster A Raviglione M Tuberculosis and HIV in people who inject drugs: evidence for action for tuberculosis, HIV, prison and harm reduction services Curr Opin HIV AIDS 2012 7 345 353 10.1097/COH.0b013e328354bd44 22678489

[B31] Clark SA Martin SL Pozniak A Steel A Ward B Dunning J Henderson DC Nelson M Gazzard B Kelleher P Tuberculosis antigen-specific immune responses can be detected using enzyme-linked immunospot technology in human immunodeficiency virus (HIV)-1 patients with advanced disease Clin Exp Immunol 2007 150 238 244 10.1111/j.1365-2249.2007.03477.x 17672869 PMC2219352

[B32] Aichelburg MC Rieger A Breitenecker F Pfistershammer K Tittes J Eltz S Aichelburg AC Stingl G Makristathis A Kohrgruber N Detection and prediction of active tuberculosis disease by a whole-blood interferon-gamma release assay in HIV-1-infected individuals Clin Infect Dis 2009 48 954 962 10.1086/597351 19245343

[B33] Elliott JH Vohith K Saramony S Savuth C Dara C Sarim C Huffam S Oelrichs R Sophea P Saphonn V Immunopathogenesis and diagnosis of tuberculosis and tuberculosis-associated immune reconstitution inflammatory syndrome during early antiretroviral therapy J Infect Dis 2009 200 1736 1745 10.1086/644784 19874177

[B34] Denkinger CM Dheda K Pai M Guidelines on interferon-gamma release assays for tuberculosis infection: concordance, discordance or confusion? Clin Microbiol Infect 2011 17 806 814 10.1111/j.1469-0691.2011.03555.x 21682801

[B35] World Health Organization. Regional Office for Europe Patient Evaluation and Antiretroviral Treatment for Adults and Adolescents: Clinical Protocol for the WHO European Region 2012 Copenhagen: WHO Regional Office for Europe

[B36] Canadian Tuberculosis Committee Recommendations on interferon gamma release assays for the diagnosis of latent tuberculosis infection - 2010 Update Can Commun Dis Rep 2010 36 1 22 10.14745/ccdr.v36i00a05 PMC6802443 31701952

[B37] LoBue PA Castro KG Is it time to replace the tuberculin skin test with a blood test? JAMA 2012 308 241 242 10.1001/jama.2012.7511 22797639 PMC4629850

[B38] de Perio MA Tsevat J Roselle GA Kralovic SM Eckman MH Cost-effectiveness of interferon gamma release assays vs tuberculin skin tests in health care workers Arch Intern Med 2009 169 179 187 10.1001/archinternmed.2008.524 19171815

